# F99. FIRST EPISODE PSYCHOSIS PATIENTS WHO USED CANNABIS DEVELOP THEIR ILLNESS AT A SIGNIFICANTLY YOUNGER AGE THAN THOSE WHO NEVER USED CONSISTENTLY ACROSS EUROPE AND BRAZIL

**DOI:** 10.1093/schbul/sby017.630

**Published:** 2018-04-01

**Authors:** Caterina La Cascia, Laura Ferraro, Fabio Seminerio, Giada Tripoli, Lucia Sideli, Diego Quattrone, Marta Di Forti, Daniele La Barbera, Robin Murray

**Affiliations:** 1 Ricercatore Universitario; 2 University of Palermo; 3 Institute of Psychiatry, King’s College London

## Abstract

**Background:**

Patients presenting to psychiatric services with their first episode of psychosis (FEP) report higher rates of previous cannabis use than the general population (Donoghue et al., 2011; Myles, Myles and Large, 2016). Evidence suggested that patients suffering from psychosis with a history of cannabis use have an earlier age of onset of psychosis (AOP) than those who never used it (Di Forti et al., 2013).

We aim to investigate if the reported association between use of cannabis and AOP is consistent across different countries, once having taken into account different patterns of cannabis use (i.e. frequency of use and age at first use).

**Methods:**

We analysed data on patterns of lifetime cannabis use and AOP from FEP=1,149 (61.7% males) from 5 European countries and Brazil part of the European network of national schizophrenia networks studying European Gene-Environment-Interaction (EUGEI) study.

Patients met ICD-10 criteria for psychosis, ascertained by using OPCRIT (McGuffin et al., 1991).

The CEQmv (Di Forti et al., 2009) further modified for the EUGEI study, was used to collect data on lifetime frequency of cannabis use (never used/used at least once but less than daily/ everyday use) and age at first use in years (then dichotomized according to mean age at first use ≤15 years or ≥16 years).

We used two ANOVAs: age of onset was used as the outcome variable and frequency of cannabis use and age of first use were respectively entered as independent predictors, along with country, gender and self-ascribed ethnicity.

**Results:**

63.3% of our sample used cannabis at least once in lifetime. Among those who used cannabis in their lifetime, mean age at first use was 16.8 years (sd=4.6) and median age was 16 years, 42.3% tried first time cannabis at 15 years or before, 57.7% at 16 years or older.

Patients who smoked cannabis on a recreational basis (mean age 29.0; contrast=5.8, CI 95% 4.3, 7.2, p<0.001) and on a daily basis (mean age 26.6; contrast=2.4, CI 95% 0.9, 3.9, p=0.001) had lower age of onset than not users patients (mean age 34.8) across all countries, once have taken into account gender and ethnicity

Only, those who started using cannabis ≤15 years had an earlier age of onset (25.5 years) than those who started at their 16 years or later (29.5 years), (F(1,683)=37.3, p<0.001). This relationship was the same across different countries (p=0.968), and independently influenced by ethnicity (F(5, 683)=2.3, p=0.03) but not by gender (p=0.057).

**Discussion:**

Our results suggest a generalizable across country and specific effect of frequency of use and early age at first cannabis use on significantly anticipate age of psychosis onset in First episode Psychosis patients.

